# Baseline Sequencing Surveillance of Public Clinical Testing, Hospitals, and Community Wastewater Reveals Rapid Emergence of SARS-CoV-2 Omicron Variant of Concern in Arizona, USA

**DOI:** 10.1128/mbio.03101-22

**Published:** 2023-01-09

**Authors:** Matthew F. Smith, Steven C. Holland, Mihyun B. Lee, James C. Hu, Nghia C. Pham, Regan A. Sullins, LaRinda A. Holland, Tianchen Mu, Alexis W. Thomas, Remington Fitch, Erin M. Driver, Rolf U. Halden, Michelle Villegas-Gold, Sheri Sanders, Jennifer L. Krauss, Lora Nordstrom, Mary Mulrow, Michael White, Vel Murugan, Efrem S. Lim

**Affiliations:** a Center for Fundamental and Applied Microbiomics, Biodesign Institute, Arizona State University, Tempe, Arizona, USA; b Center for Personalized Diagnostics, Biodesign Institute, Arizona State University, Tempe, Arizona, USA; c Center for Environmental Health Engineering, Biodesign Institute, Arizona State University, Tempe, Arizona, USA; d Knowledge Enterprise, Arizona State University, Tempe, Arizona, USA; e Dignity Health, St. Joseph's Hospital and Medical Center, Phoenix, Arizona, USA; f Division of Pathology and Laboratory Medicine, Phoenix Children's Hospital, Phoenix, Arizona, USA; g Valleywise Health Medical Center, Phoenix, Arizona, USA; h School of Life Sciences, Arizona State University, Tempe, Arizona, USA; Medical School, National and Kapodistrian University of Athens

**Keywords:** emergence dynamics, hospital-associated mutations, Omicron variant, SARS-CoV-2, wastewater surveillance

## Abstract

The adaptive evolution of SARS-CoV-2 variants is driven by selection for increased viral fitness in transmissibility and immune evasion. Understanding the dynamics of how an emergent variant sweeps across populations can better inform public health response preparedness for future variants. Here, we investigated the state-level genomic epidemiology of SARS-CoV-2 through baseline genomic sequencing surveillance of 27,071 public testing specimens and 1,125 hospital inpatient specimens diagnosed between November 1, 2021, and January 31, 2022, in Arizona. We found that the Omicron variant rapidly displaced Delta variant in December 2021, leading to an “Omicron surge” of COVID-19 cases in early 2022. Wastewater sequencing surveillance of 370 samples supported the synchronous sweep of Omicron in the community. Hospital inpatient COVID-19 cases of Omicron variant presented to three major hospitals 10.51 days after its detection from public clinical testing. Nonsynonymous mutations in nsp3, nsp12, and nsp13 genes were significantly associated with Omicron hospital cases compared to community cases. To model SARS-CoV-2 transmissions across the state population, we developed a scalable sequence network methodology and showed that the Omicron variant spread through intracounty and intercounty transmissions. Finally, we demonstrated that the temporal emergence of Omicron BA.1 to become the dominant variant (17.02 days) was 2.3 times faster than the prior Delta variant (40.70 days) or subsequent Omicron sublineages BA.2 (39.65 days) and BA.5 (35.38 days). Our results demonstrate the uniquely rapid sweep of Omicron BA.1. These findings highlight how integrated public health surveillance can be used to enhance preparedness and response to future variants.

## INTRODUCTION

Severe acute respiratory syndrome coronavirus 2 (SARS-CoV-2) is the etiological agent of the Coronavirus Disease 2019 (COVID-19) pandemic that emerged in December 2019 (1, 2). SARS-CoV-2 has evolved variants of concern (VOC) harboring mutations that have increased transmissibility, and potential to mitigate therapeutics, vaccines, and diagnostics (3–10). In particular, Delta and Omicron VOCs were the first lineages to rise to become dominant circulating variants worldwide. The Delta variant evolved mutations that confer partial escape from neutralizing monoclonal and polyclonal antibodies, as well as exhibit characteristics of increased disease severity (4, 6, 11). The Omicron variant has increased transmissibility, considerable escape from neutralizing antibodies, and reduced disease severity relative to Delta (8, 12, 13). Omicron accrued at least 55 mutations relative to the initial Wuhan reference sequence, 30 of which are in the Spike gene. In contrast, Delta had 34 mutations relative to the Wuhan sequence, 8 of which are located in the Spike (14). The combination of Spike mutations in the Omicron variant resulted in a major immune-evasive antigenic shift in SARS-CoV-2 (15, 16). Although BA.1 and BA.2 lineages have both been designated the Omicron VOC, phylogenetic studies support that BA.1 and BA.2 branched off independently to form monophyletic (13, 17). As the global transmission of SARS-CoV-2 continues, selective pressures exerted by host immunity and vaccines are among the factors that can drive the adaptive evolution of future variants (18, 19).

Next-generation sequencing provides important insights into the evolutionary trajectory of circulating SARS-CoV-2 lineages. Genomic sequencing data can be used to improve diagnostic assays (20, 21) and vaccines (22, 23) and inform outbreak investigations. Although studies comparing COVID-19 clinical outcome typically stratify by SARS-CoV-2 lineage infections, much less is known about mutations associated with severe clinical outcomes (24). Baseline sequencing provides a representative overview of the virus lineages circulating in a geographic region. This is done by random selection of positive diagnostic specimens in a manner representative of the community demographic and clinical disease. In comparison, targeted sampling efforts such as selecting for S-gene target failure specimens or localized outbreak investigations can bias the representation of lineages circulating (25, 26). Hence, sequencing surveillance plays an integral role in enhancing public health efforts to the pandemic.

Wastewater-based epidemiology (WBE) is an effective public health approach to monitoring infectious diseases in the community (27). The advantages of WBE in the COVID-19 pandemic include estimating disease burden despite decreased clinical testing rates. Moreover, presymptomatic and asymptomatic infections may not be tested (28–30). SARS-CoV-2 wastewater sequencing reflects lineages circulating within a community (31). SARS-CoV-2 wastewater-based genomic surveillance can be used in early detection of emerging variants and virus spread that might be missed by clinical surveillance (32–35). Thus, WBE and clinical surveillance are complementary approaches to public health efforts.

Understanding the dynamics of how new SARS-CoV-2 variants emerge and sweep across the population is of critical importance to formulating informed public health responses to the ongoing pandemic. Here, we investigated the emergence of the SARS-CoV-2 Omicron variant through baseline sequencing surveillance of public clinical cases (*n *= 27,071) and hospital inpatient cases (*n *= 1,125) in Arizona at daily resolution between November 1, 2021, and January 31, 2022. We show that Omicron cases presented at hospitals 10.51 days after its emergence in community cases. We identified 4 nonsynonymous mutations in Omicron significantly associated with hospitalization compared to community cases. Wastewater genomic surveillance of Omicron showed a near-synchronous sweep in the community. We demonstrate that in Arizona, Omicron BA.1 rose to become the dominant variant within 17.02 days—more than 2-fold faster than the prior Delta variant (40.70 days), or subsequent Omicron sublineages BA.2 (39.65 days) and BA.5 (35.38 days).

## RESULTS

### Omicron BA.1 displacement of Delta variant leading to surge in COVID-19 cases.

**FIG 1:**
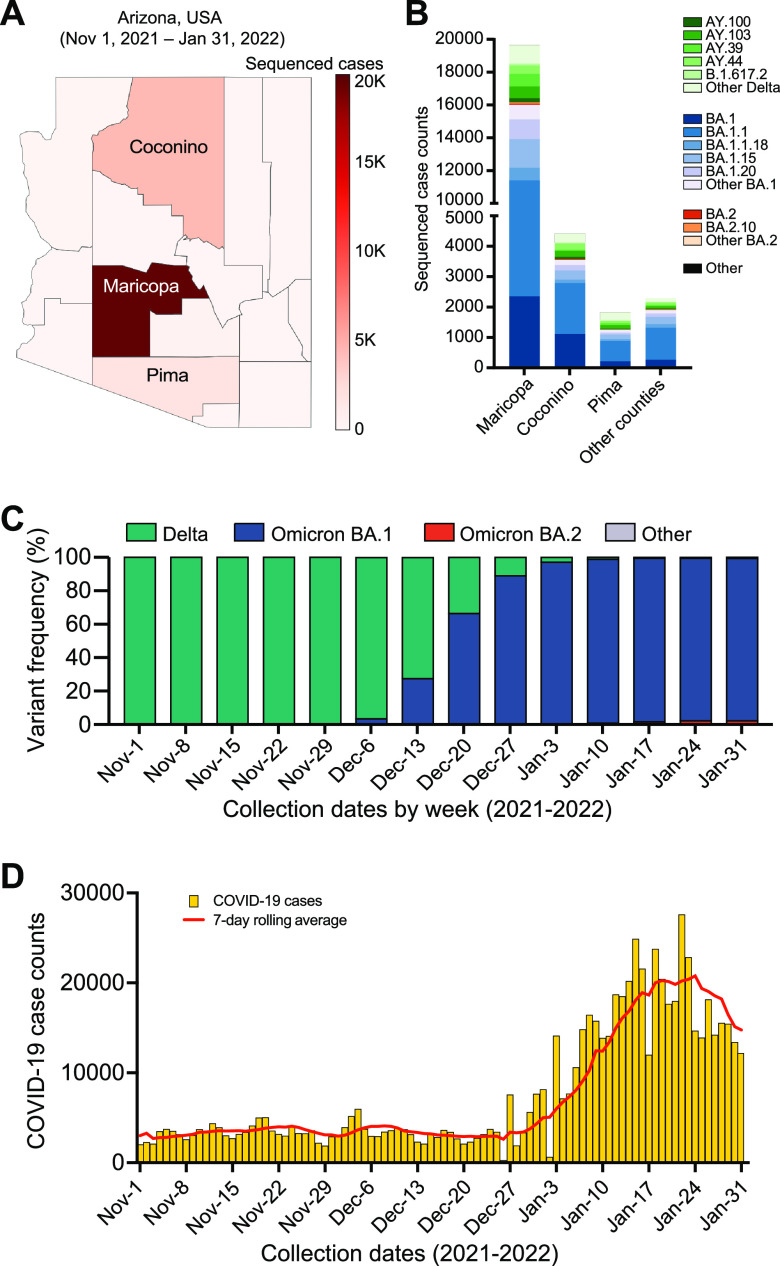
SARS-CoV-2 variants circulating in Arizona from November 1, 2021 – January 31, 2022. (A) Arizona county map showing the geographic distribution of specimens sequenced for baseline surveillance. (B) Sublineage frequencies of circulating SARS-CoV-2 genomes shown by Arizona counties. (C) Circulating SARS-CoV-2 variants in Arizona detected through community and hospital baseline surveillance shown by week. (D) Number of COVID-19 cases per day in Arizona, USA. The map in panel A was generated iwth the open source software package plotly.

As part of public health surveillance, we conducted SARS-CoV-2 baseline sequencing surveillance of SARS-CoV-2 positive diagnostic specimens collected between November 1, 2021, and January 31, 2022, in Arizona. Saliva specimens from voluntary, self-scheduled COVID-19 testing were submitted to the clinical testing laboratory from local and public testing sites across the state (Fig. 1A). In addition, positive specimens from three hospitals in the Arizona health system (Dignity Health, Phoenix Children’s Hospital, and Valleywise Health Medical Center) were submitted for sequencing surveillance. Next-generation sequencing was performed on all positive specimens without sampling bias (i.e., baseline surveillance; not targeted by prescreening for variants, travel history, disease severity, etc.). SARS-CoV-2 genome sequences were generated from 27,071 community cases (average 294 specimens per day) and 1,125 from hospital inpatient cases (average 12 specimens per day) (Table 1). Most of the sequenced specimens were collected from Maricopa County (*n *= 19,655; 69.7%), with Coconino (*n *= 4,419; 15.7%) and Pima (*n *= 1,830; 6.5%) counties having the second and third highest density of sequenced specimens, respectively (Fig. 1B). All other Arizona counties had fewer than 400 sequenced specimens, and 1,618 (5.7%) specimens were collected from patients that registered residence outside Arizona despite sample collection and testing occurring within Arizona. The prevalence of Omicron in baseline surveillance increased from 3.3% of sequenced cases the week of December 6, 2021, to 89.0% of sequenced cases the week of December 27^th^, 2021 (Fig. 1C). Following the shift to Omicron variant, Arizona experienced a surge of COVID-19 case counts beginning on December 27, 2021, peaking at 27,681 cases on January 22, 2022 (Fig. 1D).

**TABLE 1:** Participant demographics of community public testing and hospital inpatient testing cohorts

Metadata	Comprehensive demographics	Community public testing	Hospital inpatient testing
Total Samples	28,196	27,071	1,125
Age Range	5 days −110 (97) yrs	2 yrs −110 (95) yrs	5 days −97 yrs
Avg Age	31.06 yrs	30.89 yrs	35.38 yrs
No. in Range (Under 15 yrs)	2,190 (7.77%)	1,869 (6.90%)	321 (28.5%)
No. in Range (15 to 64 yrs)	24,867 (88.19%)	24,331 (89.88%)	536 (47.6%)
No. in Range (Over 65 yrs)	1,057 (3.75%)	848 (3.13%)	209 (18.6&)
Age Information Withheld	82 (0.29%)	23 (0.08%)	59 (5.24%)
No. of Males (%)	14,656 (51.98%)	14,256 (52.66%)	400 (35.56%)
No. of Females (%)	13,063 (46.33%)	12,660 (46.77%)	403 (35.82%)
Gender Information Withheld (%)	477 (1.69%)	155 (0.57%)	322 (28.62%)

### Distinct viral genomic characteristics of hospital Omicron cases.

**FIG 2:**
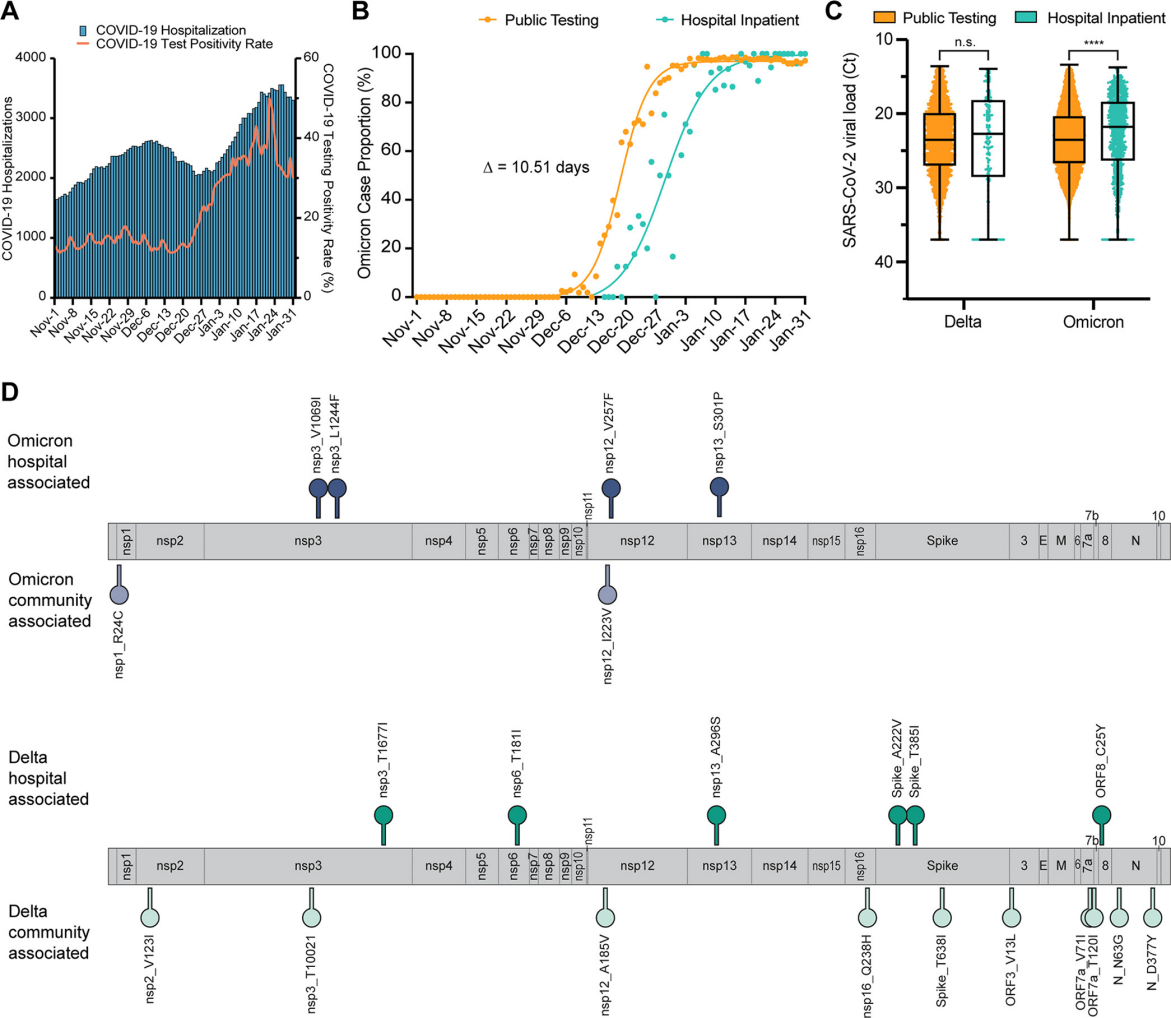
Characteristics of SARS-CoV-2 hospital surveillance. (A) Number of COVID-19 hospitalizations and reported COVID-19 diagnostic test positivity rate in Arizona. (B) Prevalence of Omicron variant in community and hospital surveillance samples. Nonlinear (sigmoidal) curves were fitted to data. The number of days between curve midpoints (50% proportion) is shown. (C) SARS-CoV-2 viral load, as measured by diagnostic RT-PCR, between Delta and Omicron variants for community and hospital surveillance samples. Statistical significance was assessed by Mann-Whitney U test. (D) SARS-CoV-2 genome map shows locations of amino acid substitutions found to be statistically associated (*P* < 0.05) with hospital or community specimens for Delta (top) and Omicron (bottom) variants. Statistical significance was assessed by Fisher’s exact test with multiple testing correction using the Benjamini/Hochberg algorithm.

In Arizona, the “Omicron surge” in COVID-19 cases was reflected in a marked increase in COVID-19 hospitalizations (Fig. 2A). To compare the temporal emergence of Omicron in the community to hospital admissions, we applied nonlinear regression modeling to the daily Omicron case proportions in each cohort, respectively, and determined the date at which Omicron accounted for 50% of case proportions. We found that Omicron first emerged in community cases, followed by hospital cases 10.51 days later (Fig. 2B). To assess whether there were differences in specimen viral loads, we compared the diagnostic CT values between community and hospital cases. Although there was no statistical difference in CT values between Delta variant specimens, the CT values of Omicron hospital specimens were significantly lower than public specimens (Mann-Whitney U test, *P* < 0.0001) suggesting that hospitalized cases had a higher viral load (Fig. 2C). While hospital rates are higher in unvaccinated individuals (36), we reasoned that there might also be specific mutations associated with hospital cases compared to community population. We identified seven nonsynonymous mutations in the Delta variant nsp3, nsp6, nsp13, S, and ORF8, that were significantly associated with hospital specimens (Fig. 2D, Fisher’s exact test with Benjamini-Hochberg multiple testing correction; Table 2). We identified four mutations in the Omicron variant nsp3, nsp12, and nsp13 that were significantly enriched in hospital specimens (Fig. 2D, Fisher’s exact test with Benjamini-Hochberg multiple testing correction; Table 3).

**TABLE 2:** Delta variant associated polymorphisms

Polymorphism	Odds ratio	Fisher’s *P* value	Corrected *P* value^*a*^
Delta hospital associated			
ORF8_C25Y	10.29	*P* < 0.001	*P* < 0.001
nsp13_A296S	2.30	0.001	0.002
Spike_T385I	3.35	0.002	0.003
nsp3_T1677I	2.84	0.007	0.012
nsp6_T181I	2.61	0.012	0.017
Spike_A222V	2.53	0.015	0.020
nsp13_I334V^*b*^	1.45	0.04	0.05
Delta community associated			
nsp3_T1002I	8.94	*P* < 0.001	*P* < 0.001
ORF3_V13L	8.81	*P* < 0.001	*P* < 0.001
N_D63G	51.05	*P* < 0.001	*P* < 0.001
nsp12_A185V	5.40	*P* < 0.001	*P* < 0.001
ORF7a_V71I	3.48	*P* < 0.001	*P* < 0.001
nsp16_Q238H	2.99	*P* < 0.001	*P* < 0.001
N_D377Y	30.55	0.001	0.001
Spike_T638I	9.81	0.001	0.003
nsp2_V123I	5.32	0.004	0.007
ORF7a_T120I	10.35	0.02	0.03

a After Benjamini-Hochberg multiple testing correction.

b Polymorphism was significant (*P* < 0.05) before multiple testing correction but is no longer significant after correction.

**TABLE 3:** Omicron variant associated polymorphisms

Polymorphism	Odds ratio	Fisher’s *P* value	Corrected *P* value^*a*^
Omicron hospital associated			
nsp13 S301P	3.17	*P* < 0.001	*P* < 0.001
nsp3 V1069I	1.76	0.001	0.005
nsp3 L1244F	2.63	0.002	0.007
nsp12 V257F	2.97	0.004	0.012
M Q19E^*b*^	6.75	0.022	0.054
M D3G*‡*	6.70	0.022	0.054
Omicron community associated			
nsp1 R24C	6.95	*P* < 0.001	*P* < 0.001
nsp12 I223V	3.67	*P* < 0.001	*P* < 0.001

a After Benjamini-Hochberg multiple testing correction.

b Polymorphism was significant (*P* < 0.05) before multiple testing correction, but is no longer significant.

### Network analysis models intracounty and intercounty transmission patterns.

**FIG 3:**
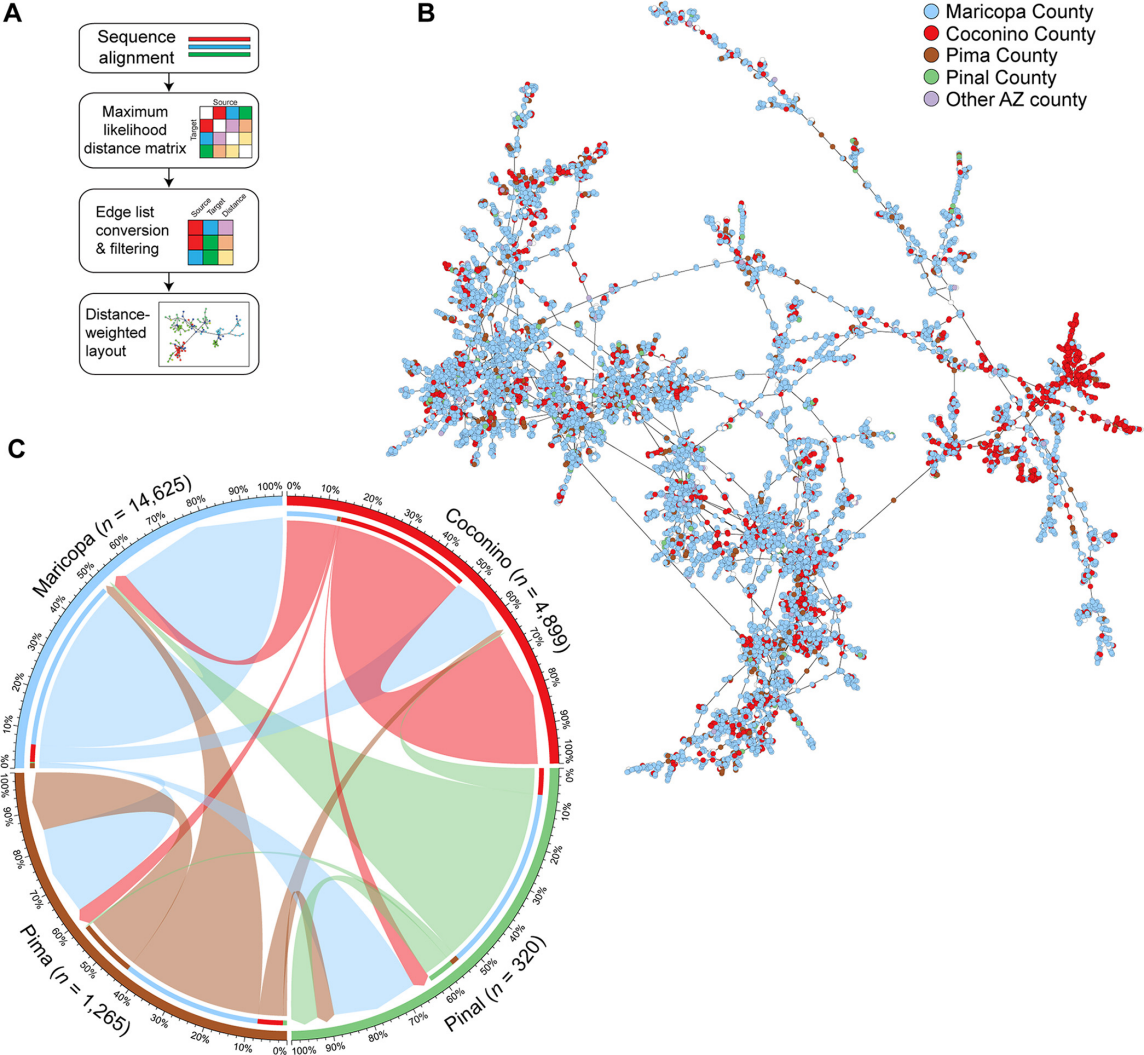
Network transmission analysis of SARS-CoV-2 genomes. (A) Overview of the bioinformatic workflow used to generate transmission networks. (B) Visualization of the largest connected Omicron BA.1 cluster of the generated network. Nodes are colored by county, generated from participant provided zip code. (C) Incoming and outgoing edges from the four most populous counties in the entire Omicron network is shown. Edges are colored by their source county and have arrowheads directed toward their target county. Outgoing edges contain an inset bar colored by the county of the targeted node.

We sought to understand the transmission dynamics of the Omicron’s emergence at the population scale. Existing tools for genomic sequence network analyses do not efficiently scale to large numbers of sequences and are particularly computationally intensive for the large genome size of SARS-CoV-2 (29.9 kb) (37). Therefore, we developed a scalable sequence network analysis methodology (described in Methods). In brief, genome sequences are aligned to the Wuhan1 reference, following which pairwise genetic distances are calculated between all samples and converted to an edge list for network visualization (Fig. 3A). A consideration of this approach is missing data from individuals who did not test and specimens that were not sequenced. For example, single source individuals absent from the network that seeded infections in multiple counties may appear as intercounty transmissions in the network. Hence, to refine transmission relationships, connections (edges) between specimens (nodes) were required to be within 10 days of collection date and only the shortest source-edge is maintained. In other words, the network showed cases in relation to their most-recent potential source of transmission. This resulted in a network from 17,734 Omicron sequence nodes and inference of 21,145 connection edges within the network (average neighbor count of 2.4). The largest Omicron network cluster contained 11,258 sequences (Fig. 3B). To validate the methodology, we assessed the network by overlaying PANGO lineage classifications and found that clusters grouped by sequence similarity (Fig. S1). We identified large plausible transmission clusters originating from and localized to Maricopa and Coconino counties. Our model suggested that substantial intracounty transmissions occurred within each county, particularly in Maricopa and Coconino counties (Fig. 3C). Further, intercounty transmission in Pima and Pinal counties could feasibly be traced to cases from Maricopa County. Overall, even when biased toward connections with the shortest physical distance, this model illustrates that robust intercounty transmission contributed in part to the spread of the Omicron variant.

10.1128/mbio.03101-22.1 FIG S1 Network transmission analysis of SARS-CoV-2 Omicron genomes by lineage. The entire Omicron network is shown. Nodes (specimens) are colored by SARS-CoV-2 lineage as classified by PANGO. Download FIG S1, TIF file, 2.2 MB. Copyright © 2023 Smith et al. 2023 Smith et al. https://creativecommons.org/licenses/by/4.0/ This content is distributed under the terms of the Creative Commons Attribution 4.0 International license.

### SARS-CoV-2 wastewater sequencing surveillance corroborates synchronous sweep of Omicron.

**FIG 4:**
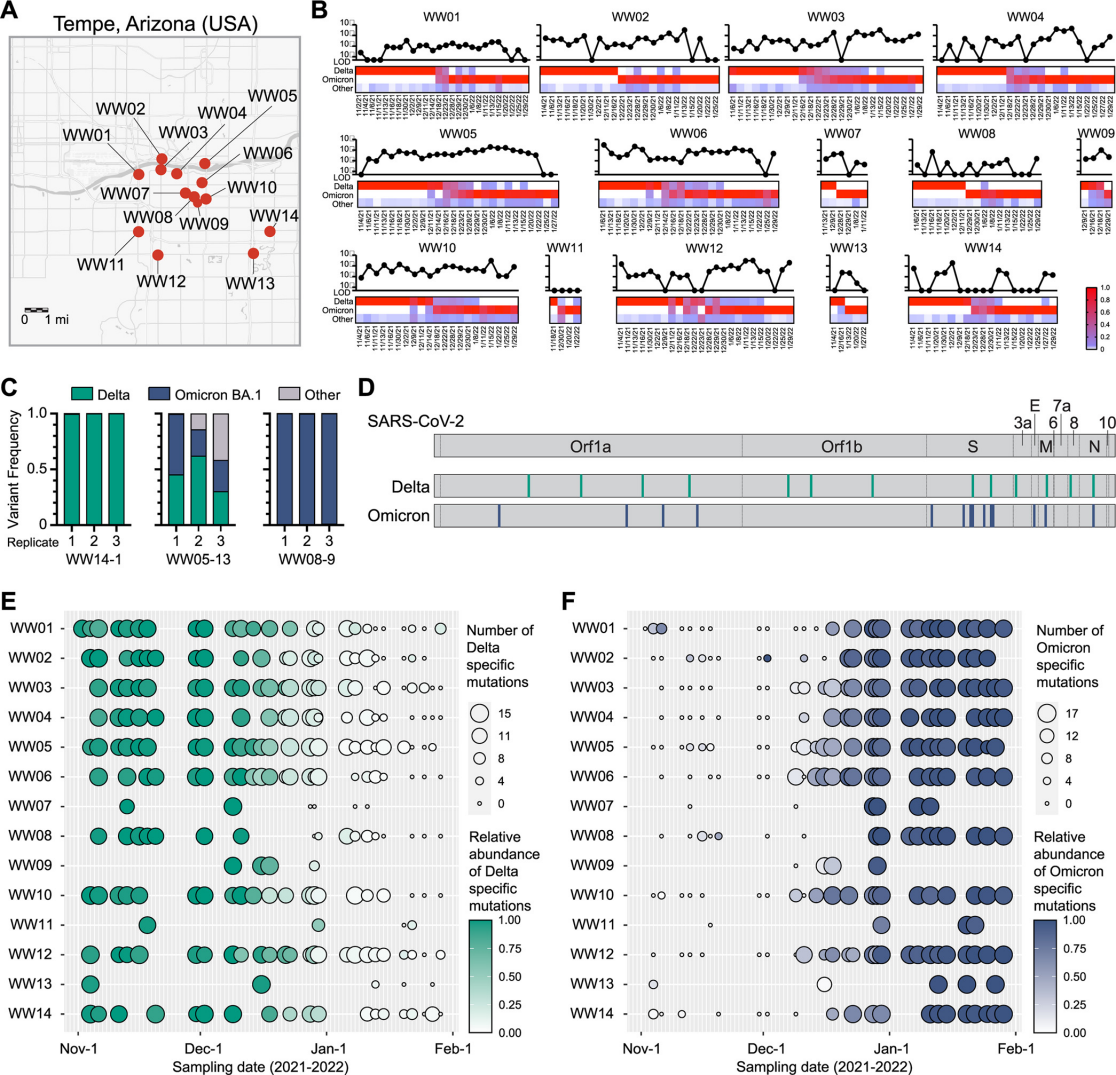
Wastewater surveillance detected Omicron variant sweeping through the community. (A) Map of wastewater collection sites monitored in the Greater Tempe area, Arizona. (B) SARS-CoV-2 viral load of wastewater samples measured by RT-PCR (line charts) and variant abundance of next generation sequencing data determined by Freyja (heatmaps) is shown for each collection site over time. (C) Representative technical replicates of next generation sequencing and variant analysis (Freyja) from three wastewater samples. Three independent extractions were performed on the wastewater samples. (D) SARS-CoV-2 genome map showing the 15 Delta- and 17 Omicron-specific mutations analyzed. (E) The number of Delta-specific mutations is indicated by the circle size, and relative abundance of the Delta-specific mutations is indicated by the color intensity for each wastewater sample. (F) The number of Omicron-specific mutations is indicated by the circle size, and relative abundance of the Omicron-specific mutations is indicated by the color intensity for each wastewater sample. The map in panel A was made using the arcGIS web interface.

Wastewater monitoring of SARS-CoV-2 is an effective surrogate of community transmission. We performed wastewater SARS-CoV-2 surveillance on 370 samples collected from 14 sites across the greater Tempe area (population of approximately 700,000) in Arizona (Fig. 4A). SARS-CoV-2 virus RNA was detected by qRT-PCR assays performed on the wastewater specimens during the study window (Fig. 4B). Genomic sequencing indicated that the Delta variant was the dominant variant throughout November and the first week of December 2021. Omicron sequences were first detected in wastewater samples obtained from two sites on December 9th, 2021. We found that the Omicron variant rapidly displaced the Delta variant to become the dominant variant typically within 1 to 2 weeks (Fig. 4B). As a control, we performed sequencing on triplicate independent wastewater extractions demonstrating consistency in wastewater variant calls by Freyja (Fig. 4C). To corroborate Freyja’s variant analysis, we compiled a list of variant specific mutations from the five most abundant sublineages of each variant observed in community samples during the investigation period. Fifteen Delta-specific and 17 Omicron-specific SNVs were found across the genome (Fig. 4D). Wastewater sequencing results were queried for the proportion of SNVs found and the mutation frequency at each locus (Fig. 4E and F). At the start of the investigation period, Delta-specific mutations are found in high prevalence and abundance, but diminished as the Omicron specific mutations increase.

**FIG 5:**
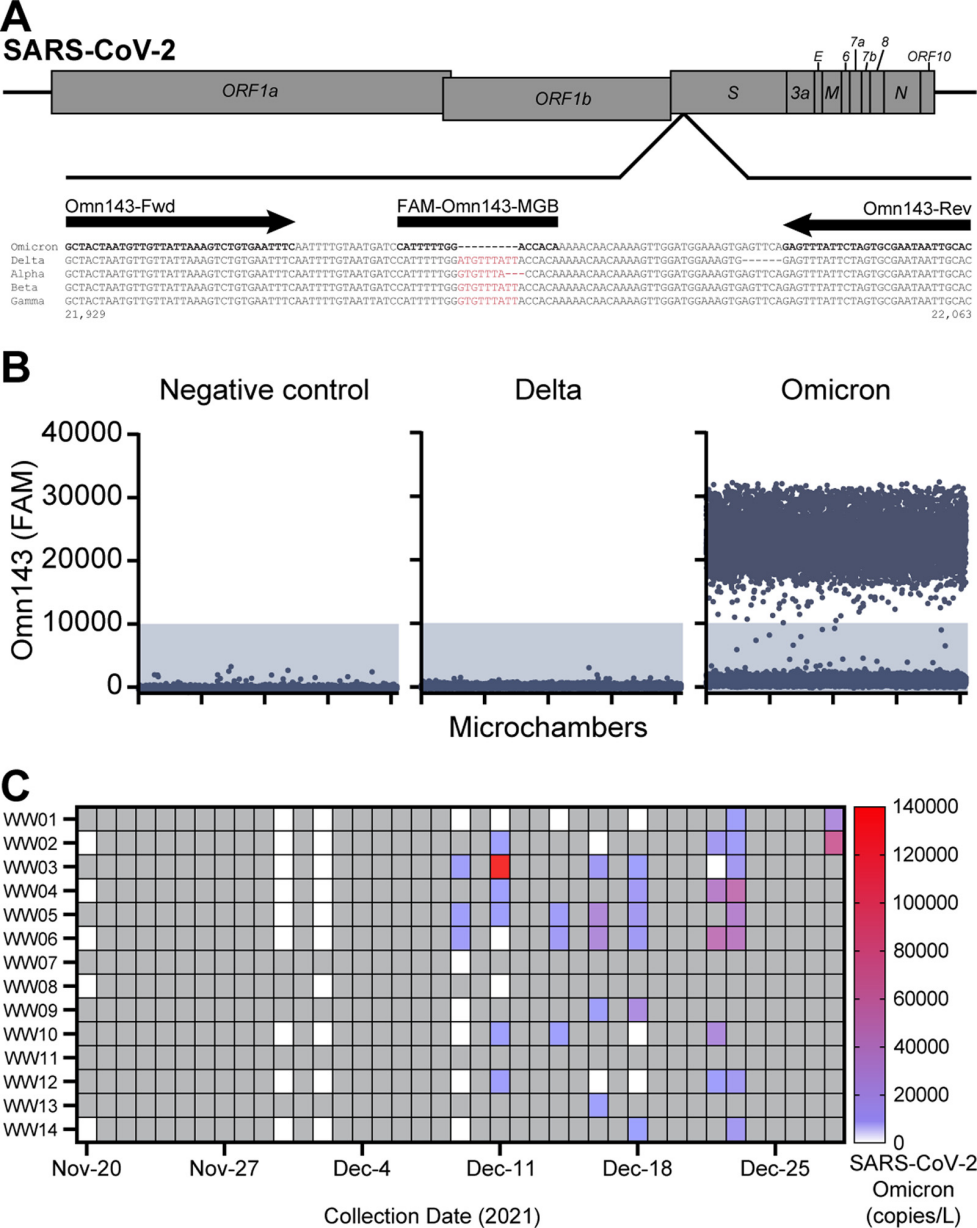
Wastewater detection of Omicron variant using a digital PCR assay. (A) The Omn143 assay design is shown, dPCR primers (arrows) and probe (box) sequences are indicated. Sequence alignment shows Omicron, Delta, Alpha, Beta and Gamma VOCs. Sequence differences in the probe binding is highlighted in red. (B) Representative dPCR results of Omn143 assay using water negative control, synthetic DNA constructs of Delta and Omicron. Three independent experiments were performed. (C) Omicron variant abundance of wastewater samples by RT-dPCR. Gray boxes indicate no sampling at that time point.

We next performed molecular validation of wastewater detection of the Omicron variant using a digital PCR (RT-dPCR) assay by leveraging a 9-nucleotide deletion unique to Omicron, but not present in other VOCs (Delta, Alpha, Beta, Gamma) (Fig. 5A). The Omn143 RT-dPCR assay specifically detected the Omicron variant sequence but not the Delta variant (Fig. 5B). Omicron variant sequences were first detected by the Omn143 RT-dPCR assay in wastewater samples on December 9th, 2021, in 3 of 9 wastewater sites sampled on that date. Between November 20th and December 28^th^, the RT-dPCR assay detected Omicron in 12 of the 14 sites, with the latest first detection for a given site occurring on December 23^rd^, 2021. Detection of Omicron sequences in wastewater by RT-dPCR coincided with detection by genomic sequencing for 29 of 43 (67.4%) wastewater samples between November 20th, 2021, and December 28^th^, 2021. RT-dPCR detected Omicron in 9 samples for which genomic sequencing did not detect Omicron within the same period, whereas genomic sequencing detected Omicron in 5 samples for which RT-dPCR did not detect Omicron. Taken together, these data supports a date of initial introduction of Omicron no later than December 9th. Further, the molecular RT-dPCR assays and genomic sequencing of wastewater samples applied in tandem expanded the capacity to detect Omicron in wastewater than either method alone.

### Omicron BA.1 displaces competing variants and rises to dominance faster than Delta and subsequent Omicron lineages.

**FIG 6:**
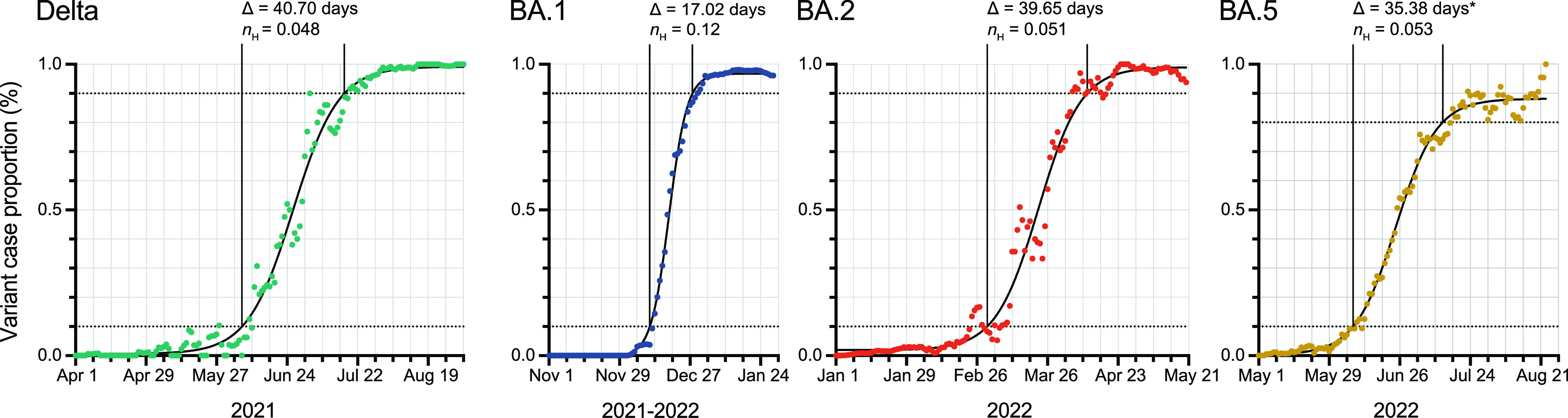
Emergence rate of Delta, Omicron BA.1, BA.2 and BA.5. Nonlinear (sigmoidal) curves were fitted for each variant using 5-day sliding window. The time it took a variant to increase from 10% to 90% case proportion (Δ) and Hillslope coefficient (*n*_H_) is indicated. (Delta: *n *= 4072, Daily Average = 26.27, *n*_H_ 95% CI = 0.045 – 0.052; Omicron BA.1: *n *= 27396, Daily Average = 304.40, *n*_H_ 95% CI = 0.11 – 0.13; Omicron BA.2: *n *= 23971, Daily Average = 171.22, *n*_H_ 95% CI = 0.046 – 0.056; Omicron BA.5: *n *= 4597, Daily Average = 39.97, *n*_H_ 95% CI = 0.050 – 0.058). Asterisk indicates calculation of emergence to 80% prevalence.

To benchmark the rate of emergence of variants to dominance, we compared Omicron BA.1 to the prior Delta, and subsequent Omicron lineages. Here, we expand the scope of genomes analyzed to include all SARS-CoV-2 baseline sequencing surveillance GISAID submissions originating from our lab between January 1st, 2021, and August 30^th^, 2022. Using nonlinear regression models of baseline sequencing surveillance data, we calculated the time it took each variant to increase from 10% to 90% case proportion in nonlinear regression models of baseline sequencing surveillance data. We show that Delta emerged and displaced other competing variants (Alpha, Beta, Gamma, Epsilon) in Arizona in 40.70 days (Fig. 6A). Omicron BA.1 emergence occurred in 17.02 days — 2.39-fold faster than Delta. Omicron BA.2 subsequently displaced BA.1 within 39.65 days—an emergence period comparable to Delta. At the time of manuscript submission, Omicron BA.5 lineages only reached a plateau of around 88% prevalence due to cocirculating Omicron BA.4. When adjusted to a window of 10% to 80% prevalence, Omicron BA.5 lineages emergence was 35.38 days, demonstrating similarity to Delta and Omicron BA.2. The hillslope value of the nonlinear regression curves provides an alternative measure of each variant’s rate of emergence accounting for differences in the prevalence plateau of each variant. Omicron BA.1’s hillslope (*n*_H_ = 0.12) is more than twice steeper than that of Delta (*n*_H_ = 0.048), BA.2 (*n*_H_ = 0.051) or BA.5 (*n*_H_ = 0.053). The 95% confidence interval for the hillslope of Delta, BA.2, and BA.5 overlap whereas the 95% confidence interval for Omicron BA.1’s hillslope excludes that of the other variants (Fig. 6A). These results corroborated the accelerated rate of emergence of Omicron BA.1. Since the introduction of Omicron BA.1 coincided with a period of seasonal holidays in the US, it is possible that social gatherings might have been an extraordinary driver of BA.1’s emergence. However, it is also possible that inherent characteristics of Omicron BA.1, such as an immune-evasive antigenic shift in Spike, might be responsible for its rate of emergence. We reasoned that an analysis of the variants from a region that is different in geography and demographics may provide insight. Hence, we compared Arizona genome sequences to those from South Africa. Indeed, we found that the rate of Omicron BA.1 emergence (13.34 days) was 3.85-fold faster than Delta (51.34 days) suggesting that Omicron BA.1 was uniquely poised for a rapid emergence (Fig. S2). Overall, Omicron BA.1 exhibited an emergence in Arizona and other locations worldwide at a timescale unmatched by competing lineages.

10.1128/mbio.03101-22.2 FIG S2 Comparison of Delta and Omicron sublineages emergence rate in South Africa. Nonlinear (sigmoidal) curves were fitted for each variant. The elapsed time between 10% prevalence and 90% prevalence of the variant in sequenced cases for the population, in 5-day sliding windows, is displayed (Δ). Hillslope coefficient (*n*_H_) is indicated. (Delta: *n *= 11748, Daily Average = 75.79, *n*_H_ 95% CI = 0.043 – 0.047; Omicron BA.1: *n *= 5346, Daily Average = 77.48, *n*_H_ 95% CI = 0.16 – 0.19; Omicron BA.2: *n *= 11018, Daily Average = 97.50, *n*_H_ 95% CI = 0.037 – 0.042; Omicron BA.5: *n *= 7491, Daily Average = 48.33, *n*_H_ 95% CI = 0.00– 0.012). Asterisk indicates calculation of emergence to 80% prevalence. Download FIG S2, EPS file, 1.4 MB. Copyright © 2023 Smith et al. 2023 Smith et al. https://creativecommons.org/licenses/by/4.0/ This content is distributed under the terms of the Creative Commons Attribution 4.0 International license.

## DISCUSSION

In summary, we traced the prominent emergence of SARS-CoV-2 Omicron variant through the community and health system in Arizona. The rate of emergence of Omicron BA.1 (17.02 days) was more than 2.3-fold faster than prior Delta variant and subsequent Omicron lineages BA.2 and BA.5. Our results reveal the uniquely rapid sweep of Omicron BA.1 in Arizona.

Since SARS-CoV-2 genomic sequencing is typically tied to specimens from clinical laboratory-based and point-of-care testing, changes in testing behavior can impact the ability to survey for new variants. Changes to social behavior, funding reimbursements for testing and perceived risks to COVID-19 have led to decreased testing rates (38). COVID-19 self-tests are increasingly used which has made individual health management more accessible (39). However, self-test results are not reported to public health agencies leading to under ascertainment of COVID-19 cases. Further, there is no formal mechanism for genomic sequencing on individuals who self-test. Conversely, individuals with more severe disease are likely to seek care from health care providers. Thus, implementation of public health SARS-CoV-2 sequencing surveillance needs to adapt accordingly.

WBE can effectively estimate COVID-19 community case prevalence (40), infer the variants in communities (32), and identify the presence of cryptic mutations (32, 35). Our wastewater sequencing and dPCR molecular assays corroborated the synchronous introduction of Omicron in the community. Interestingly, wastewater surveillance studies in AB, Canada found that the introduction of Omicron was earlier in communities closer to dense population centers and tourist attraction locations which attract international travelers (41). Similarly, the time of initial establishment and subsequent rise of Omicron in MA, USA was found to be earlier and more rapid at institutes of higher education likely owing to dense dormitory style housing and differences in social behavior (42). These findings illustrate the value of deploying wastewater surveillance strategically with on the ground local context and social structure.

Specific mutations in Spike and nonstructural genes were significantly associated with hospital cases in Omicron and Delta variants. Omicron mutations associated with hospital genomes were encoded in the Nsp13, Nsp3, and Nsp12 proteins. Nsp12 and Nsp13 are involved in viral RNA synthesis (43). Nsp3 is a viral transmembrane protein involved in the formation of double-membrane vesicles during SARS-CoV-2 replication (44). While mutations in Nsp12 and Nsp13 encoded proteins may influence viral load via involvement in viral RNA synthesis, we found no statistically significant difference in viral load between hospital cases possessing Nsp13 S301, Nsp3 V1069I, Nsp3 L1244F, or Nsp12 V257 and hospital cases with the reference amino acid at that locus (Fig. S3). No Omicron Spike protein mutations were found to be discordant between hospital and community samples. Conclusions regarding correlations between these mutations and COVID-19 disease severity may be subject to ascertainment bias. Since we do not have access to patient clinical outcomes or disease severity, we are unable to formally demonstrate that the mutations are linked to disease severity. Taken together, these findings merit further investigation of selective pressures other than immune evasion that may mediate SARS-CoV-2 evolution and differential disease severity in the community studied.

10.1128/mbio.03101-22.3 FIG S3 ORF1ab CT (viral load) distribution between hospital surveillance samples possessing reference or hospital enriched substitutions. The distribution of ORF1ab CT values as obtained with the TaqPath COVID-19 Combo kit assay (Applied Biosystems, Waltham, MA, USA) and two-tailed Mann-Whitney test results for hospital surveillance samples with (A) reference amino acid serine at position 301 of nsp13 (blue, *n* = 1097, median = 21.81, mean = 23.00) or amino acid substitution proline at position 301 of nsp13 (red, *n* = 28, median = 23.01, mean = 23.29), *P* = 0.8556. (B) Reference amino acid valine at position 1069 of nsp3 (blue, *n* = 1080, median = 21.86, mean = 23.02) or amino acid substitution isoleucine at position 1069 of nsp3 (red, *n* = 45, median = 21.22, mean = 22.67), *P* = 0.5086. (C) Reference amino acid leucine at position 1244 of nsp3 (blue, *n* = 1110, median = 21.81, mean = 23.02) or amino acid substitution phenylalanine at position 1244 of nsp3 (red, *n* = 15, median = 22.18, mean = 21.98), *P* = 0.6759. (D) Reference amino acid valine at position 257 of nsp12 (blue, *n* = 1115, median = 21.82, mean = 23.01) or amino acid substitution phenylalanine at position 257 of nsp12 (red, *n* = 10, median = 21.06, mean = 23.33), *P* = 0.8741. Download FIG S3, TIF file, 0.6 MB. Copyright © 2023 Smith et al. 2023 Smith et al. https://creativecommons.org/licenses/by/4.0/ This content is distributed under the terms of the Creative Commons Attribution 4.0 International license.

Baseline surveillance has immediate applications for public health preparedness. Notably, we found that the shift to Omicron in hospital admissions occurred 10.5 days after the rise in community cases. This means that sequencing surveillance can be used as one of the predictive indicators for emergency surge preparedness. This approach relies on consistent surveillance efforts, such as daily sampling resolution in this study. While Omicron infection is generally associated with less severe disease (45), a future variant with a shorter lag time between community to hospital may indicate more rapid progression to severe disease. Since functional characterization of new variants takes time, the rate of emergence can be benchmarked against Delta and Omicron. For example, an increased rate of emergence comparable to Omicron BA.1 may implicate antigenic shift, while a typical rate of emergence comparable to Delta/BA.2/BA.5 may suggest antigenic drift in relation to population immunity. Further evolution of Omicron BA.1, BA.2, BA.4, and BA.5 descendant lineages (e.g., BF.7, BA.2.75.2, BQ.1.1, BF.13, BM.1.1.1, etc.) have separately converged on a set of spike gene mutations within the RBD (S: R346, S: L452, S: N460, S: F486), potentially indicating strong positive selection for neutralization resistance and Spike fusogenicity (46). Despite this convergent evolution, no currently circulating lineage has demonstrated the either complete displacement of competing lineages or high rate of emergence as demonstrated by BA.1 in our study. The current landscape of Omicron sublineages currently exhibits similarities to that of Delta sublineages prior to the emergence of Omicron—several sublineages splintering from the parental lineage with a few demonstrating modest increases in fitness. These findings advance our understanding of the emergence dynamics of SARS-CoV-2 variants and demonstrate the importance of sequencing surveillance in public health preparedness for future variants.

## MATERIALS AND METHODS

### Study population.

This study was approved by Arizona State University, St. Joseph's Hospital and Medical Center, Phoenix Children's Hospital, and Valleywise Health Medical Center Institutional Review Boards. For public health surveillance, all specimens were deidentified. Specimens were not identified in any targeted sampling effort. Community sequences analyzed in the study were from 27,071 saliva specimens submitted for testing at the ASU Biodesign Clinical Testing Laboratory between November 1st, 2021, and January 31^st^, 2022. ASU’s clinical testing laboratory conducted diagnostic COVID-19 testing that was free to the public. Saliva specimens were submitted from local collection drop-off sites and public testing sites across the state. Hospital inpatient specimens analyzed in the study include 1,125 positive diagnostic nasopharyngeal swab specimens from Phoenix Children’s Hospital, St. Joseph's Hospital and Medical Center, and Valleywise Health Medical Center for SARS-CoV-2 genomic sequencing surveillance. RNA was extracted from 250 μL of saliva specimen within 33 h of sample receipt using the KingFisher Flex (Thermo Scientific, Waltham, MA, USA), following the manufacturer’s guidelines. Diagnostic testing was performed using TaqPath COVID-19 Combo kit assay (Applied Biosystems, Waltham, MA, USA), following the manufacturer’s guidelines.

### SARS-CoV-2 sequencing.

NGS library preparation for clinical specimens and wastewater samples was performed using the COVIDSeq Test (Illumina, San Diego, CA, USA) with ARTICv4 and ARTICv4.1 primer sets (47). Libraries were sequenced on the Illumina NextSeq2000 instrument using 2 × 109 paired end reads. Sequencing reads adapter sequences were trimmed using trim-galore, aligned to the Wuhan1 reference genome (MN908947.3) using the Burrows–Wheeler aligner, BWA-MEM version 0.7.17-r1188 (48), and had their primer sequences trimmed using iVAR version 1.3.1 (49). Lineage calling for community and hospital-derived sequencing data were performed with pangolin software (50), with its assignment and designation libraries up to date at the time of analysis. Sequence quality was validated and annotated using VADR version 1.4 (51). Relative SARS-CoV-2 lineage and variant abundance analysis with Freyja (32) was performed only for wastewater samples that achieved ≥20 depth for ≥70% of positions across the reference genome.

### Variant nonlinear regression analysis.

Nonlinear regression of variant emergence was performed in GraphPad Prism Version 9.4.0 for Mac (GraphPad Software, San Diego, CA, USA), using the Sigmoidal, 4PL, X is log(concentration) model with no special handling of outliers, least-squares regression fitting method, and no weighting. For the Arizona rate of emergence experiment, SARS-CoV-2 GISAID submissions were downloaded using text and collection parameters. Text: “Arizona State University.” Collection: 2021-01-12 to 2022-07-27. A total of 50,821 Arizona State University GISAID submissions were downloaded on 2022-07-27 matching these criteria, and 2,578 submissions corresponding to hospital specimens were removed from this set using internal specimen IDs. A second GISAID submission download to capture the latest BA.5 submissions was performed on 2022-08-30, using the text and collection parameters. Text: “Arizona State University.” Collection: 2021-05-01 to 2022-08-30. 5,204 sequences Arizona State University GISAID submissions were downloaded on 2022-08-30 matching these criteria from which 607 hospital specimens were removed. For the rate of emergence control experiment, SARS-CoV-2 GISAID submissions were downloaded using the location and collection parameters. Location: Africa/South Africa. Collection: 2021-01-12 to 2022-07-27. 36,802 South Africa GISAID submissions were downloaded on 2022-08-07 matching these criteria, and 16 submissions were removed from this set due to incomplete collection date. A second GISAID submission download to capture the latest BA.5 submissions was performed on 2022-08-30, using the location and collection parameters. Location: Africa/South Africa. Collection: 2022-02-18 to 2022-08-30. 8,694 South Africa GISAID submissions were downloaded matching these criteria 5-day sliding averages for each variant (Delta, BA.1, BA.2, BA.5) were calculated for the range of collection dates and were utilized for regression modeling.

### Mutation frequency comparisons.

Variant mutation frequencies for each genome sequence were tabulated from GISAID annotation metadata. Comparisons between community and hospital abundance were performed using Fisher’s exact test for mutations found in 5% or more of samples. In order to confirm sequence ambiguity was not causing mutations to not be annotated, mutations found to be significantly different between cohorts (*α* = 0.05) had their sequences confirmed for nonambiguous nucleotides at the mutation locus. Sites with a high frequency of ambiguous nucleotide calls were omitted from the analysis due to insufficient sequences (i.e., fewer than 10% of sequences remained after quality filter). Nucleotide frequencies at SNP loci were counted on wastewater bam files using Geneious Prime build 2022-07-07. Mutation abundances were adjusted to remove samples containing ambiguities. Comparisons between adjusted community and hospital abundances were performed using Fisher’s exact test with *P*-values corrected using the Benjamini/Hochberg algorithm for multiple testing correction.

### Network analysis.

To generate viral transmission networks, genome sequences were first aligned using MAFFT software version 7.490 with −6merpair and –addfragments arguments, while specifying the Wuhan1 genome (MN908947.3) as the reference genome. A maximum likelihood distance matrix was created using IQTREE2 version 2.2.0.3, and by specifying a transition substitution model, empirically determined base frequencies, and the FreeRate rate heterogeneity model (-m TIM+F+R argument). The distance matrix was converted to an edge list, and source/target pairs exceeding a genetic distance of 0.00006 were removed. For each node, the most likely source node was determined by first finding the smallest genetic distance between all connected nodes and removing edges with distances exceeding that value. Next, the difference in collection dates between all connected nodes for each target node was calculated. Edges for nodes with collection dates greater than the minimum difference were removed, as well as nodes with collection dates greater than 10 days apart. Next, the Haversine distances between zip codes for each source and target node was calculated and all edges greater than the minimum calculated distance were removed.

The network layout was arranged in Cytoscape (V 3.9.1) using a prefuse force directed layout with genetic distance as edge weights.

### Lineage specific mutations.

Lineage specific mutations for each variant were compiled from the five most abundant sublineages of each variant found in community samples over the study period (Table S1). Mutation prevalence from each sublineage was obtained from Outbreak.info (52, 53). To be considered a lineage specific mutation, mutations were required to have prevalence greater than 95% in one variant and less than 1% in the other. Mutation frequency for each wastewater sample was calculated from variant files created using iVAR (V 1.3.1) with a minimum base quality filter of 20 and the Wuhan1 genome (MN908947.3) as a reference.

10.1128/mbio.03101-22.4 TABLE S1 Variant lineages and specific mutations used to validate Freyja analysis. Download Table S1, DOCX file, 0.01 MB. Copyright © 2023 Smith et al. 2023 Smith et al. https://creativecommons.org/licenses/by/4.0/ This content is distributed under the terms of the Creative Commons Attribution 4.0 International license.

### Wastewater sample collection, processing, and analysis.

Three hundred seventy composite samples of untreated wastewater were collected over 24 h from within the wastewater collection system using high frequency automated samplers at 14 locations in the Greater Tempe area, Arizona (population of approximately 700,000), between November 2, 2021, and January 29, 2022. Samples were transferred to 1L high-density polyethylene (HDPE) bottles and stored at 4°C until nucleic acid extraction. Wastewater samples were processed as previously described (31). Briefly, raw wastewater was filtered through a 0.45 μm polyethersulfone (PES) membrane filter (Fisher Scientific, Lenexa, KS, USA). Viruses present in the resultant solids-depleted filtrate were concentrated on an Amicon ultra 15 centrifugal filter with a 10,000 molecular weight cutoff (MWCO) (Millipore Sigma, Burlington, MA, USA). RNA was extracted from the concentrate using a Qiagen RNeasy minikit (Qiagen, Germantown, MD, USA). Reverse transcription–quantitative PCR (RT-qPCR) was performed on an Applied Biosystems QuantStudio 3 real-time PCR system using SuperScriptIII One-Step RT-PCR System with Platinum *Taq* DNA polymerase (Invitrogen, Carlsbad, CA, USA) and the Charité/Berlin (World Health Organization) protocol primer and probe E (envelope) gene target.

### Omicron RT-dPCR assay.

The Omicron RT-dPCR assays (Omn143) was performed using the QuantStudio Absolute Q Digital PCR System (Applied Biosystems, Waltham, MA, USA) in 10 μL reaction mixtures (including 10% overage) containing 2.5 μL of 4× Combinati one-step RT-dPCR MasterMix, 0.4 μL of each 10 μM primer (Omn143-Fwd and Omn143-Rev; Table S2), 0.2 μL of 10 μM probe (Omn143 Probe; Table S2), and 6.5 μL of sample RNA. Reaction mixtures were loaded into QuantStudio Absolute Q MAP16 plates and overlaid with 15 μL of isolation buffer. The assay was performed with thermal cycling conditions: reverse transcription at 50°C for 10 min, activation/denaturation at 95°C for 5 min, followed by 45 cycles of denaturation at 95°C for 5 s and annealing/extension at 54°C for 15 s. Assay validation was performed in three independent experiments using synthesized gBlock gene fragments (Integrated DNA Technologies, Coralville, IA, USA): Omicron BA.1 control and Delta control (Table S2). Microreaction chambers that passed QC, as determined by ROX signal, were analyzed. FAM fluorescence intensity threshold to call Omicron positive detection per microchamber was set at 10,000 (more than two standard deviations above water and Delta negative controls). Absolute quantification (copies) was reported.

10.1128/mbio.03101-22.5 TABLE S2 Omicron RT-dPCR assay (Omn143) components and sequences. Download Table S2, DOCX file, 0.01 MB. Copyright © 2023 Smith et al. 2023 Smith et al. https://creativecommons.org/licenses/by/4.0/ This content is distributed under the terms of the Creative Commons Attribution 4.0 International license.

### Data availability.

Code used for network analysis is available at https://github.com/ASU-Lim-Lab/Network_analysis. Genome sequences have been deposited to the GISAID repository.

## References

[1] Zhu N, Zhang D, Wang W, Li X, Yang B, Song J, Zhao X, Huang B, Shi W, Lu R, Niu P, Zhan F, Ma X, Wang D, Xu W, Wu G, Gao GF, Tan W. China Novel Coronavirus I, Research T. 2020. A novel coronavirus from patients with pneumonia in China, 2019. N Engl J Med 382:727–733. doi:10.1056/NEJMoa2001017. 31978945 PMC7092803

[2] Cucinotta D, Vanelli M. 2020. WHO Declares COVID-19 a Pandemic. Acta Biomed 91:157–160. doi:10.23750/abm.v91i1.9397. 32191675 PMC7569573

[3] Volz E, Mishra S, Chand M, Barrett JC, Johnson R, Geidelberg L, Hinsley WR, Laydon DJ, Dabrera G, O'Toole A, Amato R, Ragonnet-Cronin M, Harrison I, Jackson B, Ariani CV, Boyd O, Loman NJ, McCrone JT, Goncalves S, Jorgensen D, Myers R, Hill V, Jackson DK, Gaythorpe K, Groves N, Sillitoe J, Kwiatkowski DP, Consortium C-GU, Flaxman S, Ratmann O, Bhatt S, Hopkins S, Gandy A, Rambaut A, Ferguson NM. COVID-19 Genomics UK (COG-UK) consortium. 2021. Assessing transmissibility of SARS-CoV-2 lineage B.1.1.7 in England. Nature 593:266–269. doi:10.1038/s41586-021-03470-x. 33767447

[4] Ong SWX, Chiew CJ, Ang LW, Mak TM, Cui L, Toh M, Lim YD, Lee PH, Lee TH, Chia PY, Maurer-Stroh S, Lin RTP, Leo YS, Lee VJ, Lye DC, Young BE. 2021. Clinical and virological features of SARS-CoV-2 variants of concern: a retrospective cohort study comparing B.1.1.7 (Alpha), B.1.315 (Beta), and B.1.617.2 (Delta). Clin Infect Dis 75:e1128–e1136. doi:10.1093/cid/ciab721. PMC8522361 34423834

[5] Davies NG, Abbott S, Barnard RC, Jarvis CI, Kucharski AJ, Munday JD, Pearson CAB, Russell TW, Tully DC, Washburne AD, Wenseleers T, Gimma A, Waites W, Wong KLM, van Zandvoort K, Silverman JD, Group CC-W, Consortium C-GU, Diaz-Ordaz K, Keogh R, Eggo RM, Funk S, Jit M, Atkins KE, Edmunds WJ. CMMID COVID-19 Working Group. 2021. Estimated transmissibility and impact of SARS-CoV-2 lineage B.1.1.7 in England. Science 372:eabg3055. doi:10.1126/science.abg3055. 33658326 PMC8128288

[6] Planas D, Veyer D, Baidaliuk A, Staropoli I, Guivel-Benhassine F, Rajah MM, Planchais C, Porrot F, Robillard N, Puech J, Prot M, Gallais F, Gantner P, Velay A, Le Guen J, Kassis-Chikhani N, Edriss D, Belec L, Seve A, Courtellemont L, Pere H, Hocqueloux L, Fafi-Kremer S, Prazuck T, Mouquet H, Bruel T, Simon-Loriere E, Rey FA, Schwartz O. 2021. Reduced sensitivity of SARS-CoV-2 variant Delta to antibody neutralization. Nature 596:276–280. doi:10.1038/s41586-021-03777-9. 34237773

[7] Cele S, Gazy I, Jackson L, Hwa SH, Tegally H, Lustig G, Giandhari J, Pillay S, Wilkinson E, Naidoo Y, Karim F, Ganga Y, Khan K, Bernstein M, Balazs AB, Gosnell BI, Hanekom W, Moosa MS, Network For Genomic Surveillance i, South A, Team C-K, Lessells RJ, de Oliveira T, Sigal A. COMMIT-KZN Team. 2021. Escape of SARS-CoV-2 501Y.V2 from neutralization by convalescent plasma. Nature 593:142–146. doi:10.1038/s41586-021-03471-w. 33780970 PMC9867906

[8] Dejnirattisai W, Huo J, Zhou D, Zahradnik J, Supasa P, Liu C, Duyvesteyn HME, Ginn HM, Mentzer AJ, Tuekprakhon A, Nutalai R, Wang B, Dijokaite A, Khan S, Avinoam O, Bahar M, Skelly D, Adele S, Johnson SA, Amini A, Ritter TG, Mason C, Dold C, Pan D, Assadi S, Bellass A, Omo-Dare N, Koeckerling D, Flaxman A, Jenkin D, Aley PK, Voysey M, Costa Clemens SA, Naveca FG, Nascimento V, Nascimento F, Fernandes da Costa C, Resende PC, Pauvolid-Correa A, Siqueira MM, Baillie V, Serafin N, Kwatra G, Da Silva K, Madhi SA, Nunes MC, Malik T, Openshaw PJM, Baillie JK, Semple MG. ISARIC4C Consortium., et al. 2022. SARS-CoV-2 Omicron-B.1.1.529 leads to widespread escape from neutralizing antibody responses. Cell 185:467–484. doi:10.1016/j.cell.2021.12.046. 35081335 PMC8723827

[9] Holland SC, Bains A, Holland LA, Smith MF, Sullins RA, Mellor NJ, Thomas AW, Johnson N, Murugan V, Lim ES. 2022. SARS-CoV-2 Delta variant N gene mutations reduce sensitivity to the TaqPath COVID-19 multiplex molecular diagnostic assay. Viruses 14:1316. doi:10.3390/v14061316. 35746787 PMC9228125

[10] World Health Organization. 2022. Tracking SARS-CoV-2 variants. https://www.who.int/activities/tracking-SARS-CoV-2-variants.

[11] Sigal A, Milo R, Jassat W. 2022. Estimating disease severity of Omicron and Delta SARS-CoV-2 infections. Nat Rev Immunol 22:267–269. doi:10.1038/s41577-022-00720-5. 35414124 PMC9002222

[12] Shuai H, Chan JF, Hu B, Chai Y, Yuen TT, Yin F, Huang X, Yoon C, Hu JC, Liu H, Shi J, Liu Y, Zhu T, Zhang J, Hou Y, Wang Y, Lu L, Cai JP, Zhang AJ, Zhou J, Yuan S, Brindley MA, Zhang BZ, Huang JD, To KK, Yuen KY, Chu H. 2022. Attenuated replication and pathogenicity of SARS-CoV-2 B.1.1.529 Omicron. Nature 603:693–699. doi:10.1038/s41586-022-04442-5. 35062016

[13] Viana R, Moyo S, Amoako DG, Tegally H, Scheepers C, Althaus CL, Anyaneji UJ, Bester PA, Boni MF, Chand M, Choga WT, Colquhoun R, Davids M, Deforche K, Doolabh D, Du Plessis L, Engelbrecht S, Everatt J, Giandhari J, Giovanetti M, Hardie D, Hill V, Hsiao NY, Iranzadeh A, Ismail A, Joseph C, Joseph R, Koopile L, Kosakovsky Pond SL, Kraemer MUG, Kuate-Lere L, Laguda-Akingba O, Lesetedi-Mafoko O, Lessells RJ, Lockman S, Lucaci AG, Maharaj A, Mahlangu B, Maponga T, Mahlakwane K, Makatini Z, Marais G, Maruapula D, Masupu K, Matshaba M, Mayaphi S, Mbhele N, Mbulawa MB, Mendes A, Mlisana K, et al. 2022. Rapid epidemic expansion of the SARS-CoV-2 Omicron variant in southern Africa. Nature 603:679–686. doi:10.1038/s41586-022-04411-y. 35042229 PMC8942855

[14] Jung C, Kmiec D, Koepke L, Zech F, Jacob T, Sparrer KMJ, Kirchhoff F. 2022. Omicron: what Makes the latest SARS-CoV-2 variant of concern so concerning? J Virol 96:e0207721. doi:10.1128/jvi.02077-21. 35225672 PMC8941872

[15] Cameroni E, Bowen JE, Rosen LE, Saliba C, Zepeda SK, Culap K, Pinto D, VanBlargan LA, De Marco A, di Iulio J, Zatta F, Kaiser H, Noack J, Farhat N, Czudnochowski N, Havenar-Daughton C, Sprouse KR, Dillen JR, Powell AE, Chen A, Maher C, Yin L, Sun D, Soriaga L, Bassi J, Silacci-Fregni C, Gustafsson C, Franko NM, Logue J, Iqbal NT, Mazzitelli I, Geffner J, Grifantini R, Chu H, Gori A, Riva A, Giannini O, Ceschi A, Ferrari P, Cippa PE, Franzetti-Pellanda A, Garzoni C, Halfmann PJ, Kawaoka Y, Hebner C, Purcell LA, Piccoli L, Pizzuto MS, Walls AC, Diamond MS, et al. 2022. Broadly neutralizing antibodies overcome SARS-CoV-2 Omicron antigenic shift. Nature 602:664–670. doi:10.1038/s41586-021-04386-2. 35016195 PMC9531318

[16] Planas D, Saunders N, Maes P, Guivel-Benhassine F, Planchais C, Buchrieser J, Bolland WH, Porrot F, Staropoli I, Lemoine F, Pere H, Veyer D, Puech J, Rodary J, Baele G, Dellicour S, Raymenants J, Gorissen S, Geenen C, Vanmechelen B, Wawina-Bokalanga T, Marti-Carreras J, Cuypers L, Seve A, Hocqueloux L, Prazuck T, Rey FA, Simon-Loriere E, Bruel T, Mouquet H, Andre E, Schwartz O. 2022. Considerable escape of SARS-CoV-2 Omicron to antibody neutralization. Nature 602:671–675. doi:10.1038/s41586-021-04389-z. 35016199

[17] Berkhout B, Herrera-Carrillo E. 2022. SARS-CoV-2 Evolution: on the sudden appearance of the omicron variant. J Virol 96:e0009022. doi:10.1128/jvi.00090-22. 35293771 PMC9006888

[18] Choi B, Choudhary MC, Regan J, Sparks JA, Padera RF, Qiu X, Solomon IH, Kuo HH, Boucau J, Bowman K, Adhikari UD, Winkler ML, Mueller AA, Hsu TY, Desjardins M, Baden LR, Chan BT, Walker BD, Lichterfeld M, Brigl M, Kwon DS, Kanjilal S, Richardson ET, Jonsson AH, Alter G, Barczak AK, Hanage WP, Yu XG, Gaiha GD, Seaman MS, Cernadas M, Li JZ. 2020. Persistence and evolution of SARS-CoV-2 in an immunocompromised host. N Engl J Med 383:2291–2293. doi:10.1056/NEJMc2031364. 33176080 PMC7673303

[19] Hale VL, Dennis PM, McBride DS, Nolting JM, Madden C, Huey D, Ehrlich M, Grieser J, Winston J, Lombardi D, Gibson S, Saif L, Killian ML, Lantz K, Tell RM, Torchetti M, Robbe-Austerman S, Nelson MI, Faith SA, Bowman AS. 2022. SARS-CoV-2 infection in free-ranging white-tailed deer. Nature 602:481–486. doi:10.1038/s41586-021-04353-x. 34942632 PMC8857059

[20] Carter LJ, Garner LV, Smoot JW, Li Y, Zhou Q, Saveson CJ, Sasso JM, Gregg AC, Soares DJ, Beskid TR, Jervey SR, Liu C. 2020. Assay techniques and test development for COVID-19 diagnosis. ACS Cent Sci 6:591–605. doi:10.1021/acscentsci.0c00501. 32382657 PMC7197457

[21] Bohn MK, Mancini N, Loh TP, Wang CB, Grimmler M, Gramegna M, Yuen KY, Mueller R, Koch D, Sethi S, Rawlinson WD, Clementi M, Erasmus R, Leportier M, Kwon GC, Menezes ME, Patru MM, Singh K, Ferrari M, Najjar O, Horvath AR, Adeli K, Lippi G. 2020. IFCC interim guidelines on molecular testing of SARS-CoV-2 infection. Clin Chem Lab Med 58:1993–2000. doi:10.1515/cclm-2020-1412. 33027042

[22] Corbett KS, Edwards DK, Leist SR, Abiona OM, Boyoglu-Barnum S, Gillespie RA, Himansu S, Schafer A, Ziwawo CT, DiPiazza AT, Dinnon KH, Elbashir SM, Shaw CA, Woods A, Fritch EJ, Martinez DR, Bock KW, Minai M, Nagata BM, Hutchinson GB, Wu K, Henry C, Bahl K, Garcia-Dominguez D, Ma L, Renzi I, Kong WP, Schmidt SD, Wang L, Zhang Y, Phung E, Chang LA, Loomis RJ, Altaras NE, Narayanan E, Metkar M, Presnyak V, Liu C, Louder MK, Shi W, Leung K, Yang ES, West A, Gully KL, Stevens LJ, Wang N, Wrapp D, Doria-Rose NA, Stewart-Jones G, Bennett H, et al. 2020. SARS-CoV-2 mRNA vaccine design enabled by prototype pathogen preparedness. Nature 586:567–571. doi:10.1038/s41586-020-2622-0. 32756549 PMC7581537

[23] Polack FP, Thomas SJ, Kitchin N, Absalon J, Gurtman A, Lockhart S, Perez JL, Perez Marc G, Moreira ED, Zerbini C, Bailey R, Swanson KA, Roychoudhury S, Koury K, Li P, Kalina WV, Cooper D, Frenck RW, Jr, Hammitt LL, Tureci O, Nell H, Schaefer A, Unal S, Tresnan DB, Mather S, Dormitzer PR, Sahin U, Jansen KU, Gruber WC, Group CCT. C4591001 Clinical Trial Group. 2020. Safety and efficacy of the BNT162b2 mRNA Covid-19 vaccine. N Engl J Med 383:2603–2615. doi:10.1056/NEJMoa2034577. 33301246 PMC7745181

[24] Nakamichi K, Shen JZ, Lee CS, Lee A, Roberts EA, Simonson PD, Roychoudhury P, Andriesen J, Randhawa AK, Mathias PC, Greninger AL, Jerome KR, Van Gelder RN. 2021. Hospitalization and mortality associated with SARS-CoV-2 viral clades in COVID-19. Sci Rep 11:4802. doi:10.1038/s41598-021-82850-9. 33637820 PMC7910290

[25] Association of Public Health Laboratories. 10/07/2021 2021. Technical assistance and instructions for public health laboratories on categorizing sequence data as “baseline surveillance” for inclusion in CDC’s national SARS-CoV-2 genomic surveillance. https://www.aphl.org/programs/preparedness/Crisis-Management/Documents/Technical-Assistance-for-Categorizing-Baseline-Surveillance-Update-Oct2021.pdf.

[26] Goswami C, Sheldon M, Bixby C, Keddache M, Bogdanowicz A, Wang Y, Schultz J, McDevitt J, LaPorta J, Kwon E, Buyske S, Garbolino D, Biloholowski G, Pastuszak A, Storella M, Bhalla A, Charlier-Rodriguez F, Hager R, Grimwood R, Nahas SA. 2022. Identification of SARS-CoV-2 variants using viral sequencing for the Centers for Disease Control and Prevention genomic surveillance program. BMC Infect Dis 22:404. doi:10.1186/s12879-022-07374-7. 35468749 PMC9035976

[27] Hart OE, Halden RU. 2020. Computational analysis of SARS-CoV-2/COVID-19 surveillance by wastewater-based epidemiology locally and globally: feasibility, economy, opportunities and challenges. Sci Total Environ 730:138875. doi:10.1016/j.scitotenv.2020.138875. 32371231 PMC7175865

[28] Oran DP, Topol EJ. 2020. Prevalence of asymptomatic SARS-CoV-2 infection: a narrative review. Ann Intern Med 173:362–367. doi:10.7326/M20-3012. 32491919 PMC7281624

[29] Cevik M, Kuppalli K, Kindrachuk J, Peiris M. 2020. Virology, transmission, and pathogenesis of SARS-CoV-2. BMJ 371:m3862. doi:10.1136/bmj.m3862. 33097561

[30] Qiu X, Nergiz AI, Maraolo AE, Bogoch II, Low N, Cevik M. 2020. Defining the role of asymptomatic and pre-symptomatic SARS-CoV-2 transmission - a living systematic review. medRxiv. doi:10.1101/2020.09.01.20135194. PMC7825872 33484843

[31] Fontenele RS, Kraberger S, Hadfield J, Driver EM, Bowes D, Holland LA, Faleye TOC, Adhikari S, Kumar R, Inchausti R, Holmes WK, Deitrick S, Brown P, Duty D, Smith T, Bhatnagar A, Yeager RA, Holm RH, von Reitzenstein NH, Wheeler E, Dixon K, Constantine T, Wilson MA, Lim ES, Jiang X, Halden RU, Scotch M, Varsani A. 2021. High-throughput sequencing of SARS-CoV-2 in wastewater provides insights into circulating variants. Water Res 205:117710. doi:10.1016/j.watres.2021.117710. 34607084 PMC8464352

[32] Karthikeyan S, Levy JI, De Hoff P, Humphrey G, Birmingham A, Jepsen K, Farmer S, Tubb HM, Valles T, Tribelhorn CE, Tsai R, Aigner S, Sathe S, Moshiri N, Henson B, Mark AM, Hakim A, Baer NA, Barber T, Belda-Ferre P, Chacón M, Cheung W, Cresini ES, Eisner ER, Lastrella AL, Lawrence ES, Marotz CA, Ngo TT, Ostrander T, Plascencia A, Salido RA, Seaver P, Smoot EW, McDonald D, Neuhard RM, Scioscia AL, Satterlund AM, Simmons EH, Abelman DB, Brenner D, Bruner JC, Buckley A, Ellison M, Gattas J, Gonias SL, Hale M, Hawkins F, Ikeda L, Jhaveri H, Johnson T, et al. 2022. Wastewater sequencing reveals early cryptic SARS-CoV-2 variant transmission. Nature 609:101–108. doi:10.1038/s41586-022-05049-6. 35798029 PMC9433318

[33] Karthikeyan S, Nguyen A, McDonald D, Zong Y, Ronquillo N, Ren J, Zou J, Farmer S, Humphrey G, Henderson D, Javidi T, Messer K, Anderson C, Schooley R, Martin NK, Knight R. 2021. Rapid, Large-scale wastewater surveillance and automated reporting system enable early detection of nearly 85% of COVID-19 cases on a university campus. mSystems 6:e0079321. doi:10.1128/mSystems.00793-21. 34374562 PMC8409724

[34] Scott LC, Aubee A, Babahaji L, Vigil K, Tims S, Aw TG. 2021. Targeted wastewater surveillance of SARS-CoV-2 on a university campus for COVID-19 outbreak detection and mitigation. Environ Res 200:111374. doi:10.1016/j.envres.2021.111374. 34058182 PMC8163699

[35] Smyth DS, Trujillo M, Gregory DA, Cheung K, Gao A, Graham M, Guan Y, Guldenpfennig C, Hoxie I, Kannoly S, Kubota N, Lyddon TD, Markman M, Rushford C, San KM, Sompanya G, Spagnolo F, Suarez R, Teixeiro E, Daniels M, Johnson MC, Dennehy JJ. 2022. Tracking cryptic SARS-CoV-2 lineages detected in NYC wastewater. Nat Commun 13. doi:10.1038/s41467-022-28246-3. PMC8813986 35115523

[36] Danza P, Koo TH, Haddix M, Fisher R, Traub E, K OY, Balter S. 2022. SARS-CoV-2 Infection and hospitalization among adults aged >/=18 years, by vaccination status, before and during sars-cov-2 b.1.1.529 (omicron) variant predominance—Los Angeles County, California, November 7, 2021-January 8, 2022. MMWR Morb Mortal Wkly Rep 71:177–181. doi:10.15585/mmwr.mm7105e1. 35113851 PMC8812833

[37] Campbell EM, Boyles A, Shankar A, Kim J, Knyazev S, Cintron R, Switzer WM. 2021. MicrobeTrace: retooling molecular epidemiology for rapid public health response. PLoS Comput Biol 17:e1009300. doi:10.1371/journal.pcbi.1009300. 34492010 PMC8491948

[38] Centers for Disease Control and Prevention. 2022. November 02. COVID data tracker, Atlanta, GA: US Department of Health and Human Services, CDC. https://covid.cdc.gov/covid-data-tracker.

[39] Ritchey MD, Rosenblum HG, Del Guercio K, Humbard M, Santos S, Hall J, Chaitram J, Salerno RM. 2022. COVID-19 self-test data: challenges and opportunities—United States, October 31, 2021–June 11, 2022. MMWR Morb Mortal Wkly Rep 71:1005–1010. doi:10.15585/mmwr.mm7132a1. 35951486 PMC9400539

[40] Layton BA, Kaya D, Kelly C, Williamson KJ, Alegre D, Bachhuber SM, Banwarth PG, Bethel JW, Carter K, Dalziel BD, Dasenko M, Geniza M, George A, Girard AM, Haggerty R, Higley KA, Hynes DM, Lubchenco J, McLaughlin KR, Nieto FJ, Noakes A, Peterson M, Piemonti AD, Sanders JL, Tyler BM, Radniecki TS. 2022. Evaluation of a wastewater-based epidemiological approach to estimate the prevalence of SARS-CoV-2 infections and the detection of viral variants in disparate Oregon communities at city and neighborhood scales. Environ Health Perspect 130:67010. doi:10.1289/EHP10289. 35767012 PMC9241984

[41] Hubert CRJ, Acosta N, Waddell BJM, Hasing ME, Qiu Y, Fuzzen M, Harper NBJ, Bautista MA, Gao T, Papparis C, Van Doorn J, Du K, Xiang K, Chan L, Vivas L, Pradhan P, McCalder J, Low K, England WE, Kuzma D, Conly J, Ryan MC, Achari G, Hu J, Cabaj JL, Sikora C, Svenson L, Zelyas N, Servos M, Meddings J, Hrudey SE, Frankowski K, Parkins MD, Pang XL, Lee BE. 2022. Tracking emergence and spread of SARS-CoV-2 Omicron variant in large and small communities by wastewater monitoring in Alberta. Canada Emerg Infect Dis 28:1770–1776. 35867051 10.3201/eid2809.220476 PMC9423933

[42] Petros BA, Turcinovic J, Welch NL, White LF, Kolaczyk ED, Bauer MR, Cleary M, Dobbins ST, Doucette-Stamm L, Gore M, Nair P, Nguyen TG, Rose S, Taylor BP, Tsang D, Wendlandt E, Hope M, Platt JT, Jacobson KR, Bouton T, Yune S, Auclair JR, Landaverde L, Klapperich CM, Hamer DH, Hanage WP, MacInnis BL, Sabeti PC, Connor JH, Springer M. 2022. Early introduction and rise of the Omicron SARS-CoV-2 variant in highly vaccinated university populations. Clin Infect Dis. doi:10.1093/cid/ciac413. PMC9213864 35616119

[43] Yadav R, Chaudhary JK, Jain N, Chaudhary PK, Khanra S, Dhamija P, Sharma A, Kumar A, Handu S. 2021. Role of structural and non-structural proteins and therapeutic targets of SARS-CoV-2 for COVID-19. Cells 10. doi:10.3390/cells10040821. PMC8067447 33917481

[44] Ricciardi S, Guarino AM, Giaquinto L, Polishchuk EV, Santoro M, Di Tullio G, Wilson C, Panariello F, Soares VC, Dias SSG, Santos JC, Souza TML, Fusco G, Viscardi M, Brandi S, Bozza PT, Polishchuk RS, Venditti R, De Matteis MA. 2022. The role of NSP6 in the biogenesis of the SARS-CoV-2 replication organelle. Nature 606:761–768. doi:10.1038/s41586-022-04835-6. 35551511 PMC7612910

[45] Vijayaraghavan B, Silva R, Thwin S, Diaz J, Bertagnolio S. 2022. Severity of disease associated with Omicron variant as compared with Delta variant in hospitalized patients with suspected or confirmed SARS-CoV-2 infection. Geneva: World Health Organization.

[46] Qu P, Evans JP, Faraone J, Zheng YM, Carlin C, Anghelina M, Stevens P, Fernandez S, Jones D, Lozanski G, Panchal A, Saif LJ, Oltz EM, Xu K, Gumina RJ, Liu SL. 2022. Distinct neutralizing antibody escape of SARS-CoV-2 Omicron subvariants BQ.1, BQ.1.1, BA.4.6, BF.7 and BA.2.75.2. bioRxiv. doi:10.1101/2022.10.19.512891. PMC9678813 36476380

[47] ARTIC Network. 2022. artic-network/primer-schemes/nCOV-2019. https://github.com/artic-network/primer-schemes/tree/master/nCoV-2019/V4.

[48] Li H. 2013. Aligning sequence reads, clone sequences and assembly contigs with BWA-MEM. arXiv doi:10.48550/ARXIV.1303.3997.

[49] Castellano S, Cestari F, Faglioni G, Tenedini E, Marino M, Artuso L, Manfredini R, Luppi M, Trenti T, Tagliafico E. 2021. iVar, an Interpretation-oriented tool to manage the update and revision of variant annotation and classification. Genes (Basel):12. 33800487 10.3390/genes12030384 PMC8001268

[50] Rambaut A, Holmes EC, O'Toole A, Hill V, McCrone JT, Ruis C, Du Plessis L, Pybus OG. 2020. A dynamic nomenclature proposal for SARS-CoV-2 lineages to assist genomic epidemiology. Nat Microbiol 5:1403–1407. doi:10.1038/s41564-020-0770-5. 32669681 PMC7610519

[51] Schaffer AA, Hatcher EL, Yankie L, Shonkwiler L, Brister JR, Karsch-Mizrachi I, Nawrocki EP. 2020. VADR: validation and annotation of virus sequence submissions to GenBank. BMC Bioinformatics 21. doi:10.1186/s12859-020-3537-3. PMC7245624 32448124

[52] Gangavarapu K, Latif AA, Mullen JL, Alkuzweny M, Hufbauer E, Tsueng G, Haag E, Zeller M, Aceves CM, Zaiets K, Cano M, Zhou J, Qian Z, Sattler R, Matteson NL, Levy JI, Lee RTC, Freitas L, Maurer-Stroh S, Suchard MA, Wu C, Su AI, Andersen KG, Hughes LD. 2022. Outbreak.info genomic reports: scalable and dynamic surveillance of SARS-CoV-2 variants and mutations. medRxiv. doi:10.1101/2022.01.27.22269965. PMC10399614 36823332

[53] Tsueng G, Mullen JL, Alkuzweny M, Cano M, Rush B, Haag E, Outbreak C, Latif AA, Zhou X, Qian Z, Hufbauer E, Zeller M, Andersen KG, Wu C, Su AI, Gangavarapu K, Hughes LD. 2022. Outbreak.info research library: a standardized, searchable platform to discover and explore COVID-19 resources. bioRxiv. doi:10.1101/2022.01.20.477133. PMC10393269 36823331

