# Supplementary figures and images for: Baseline Sequencing Surveillance of Public Clinical Testing, Hospitals, and Community Wastewater Reveals Rapid Emergence of SARS-CoV-2 Omicron Variant of Concern in Arizona, USA
Source: mBio. 2023 Jan 9;14(1):e03101-22. doi: 10.1128/mbio.03101-22 (PMC9972916; doi:10.1128/mbio.03101-22)

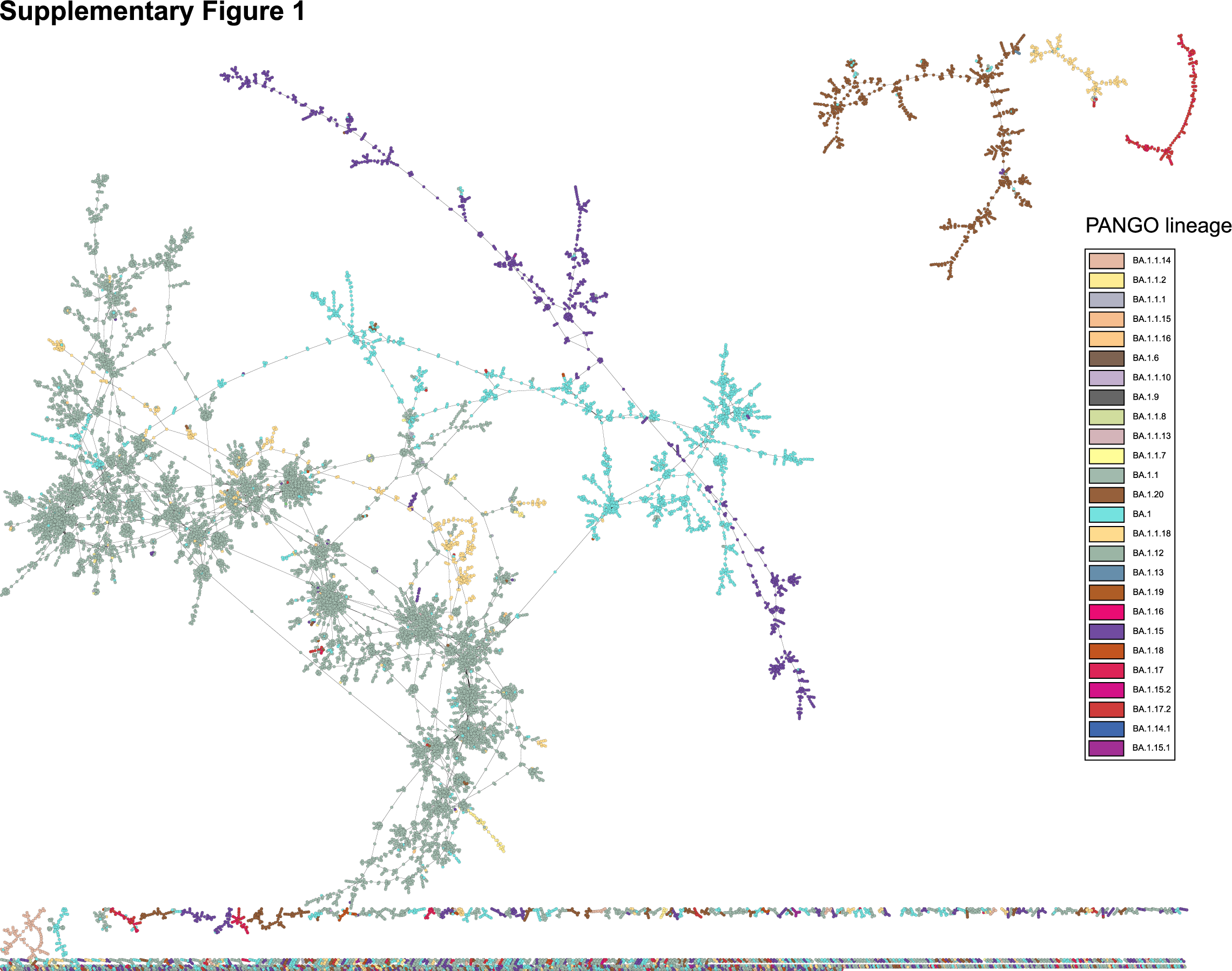

Supplement: FIG S1 [file mbio.03101-22-s0001.tif]

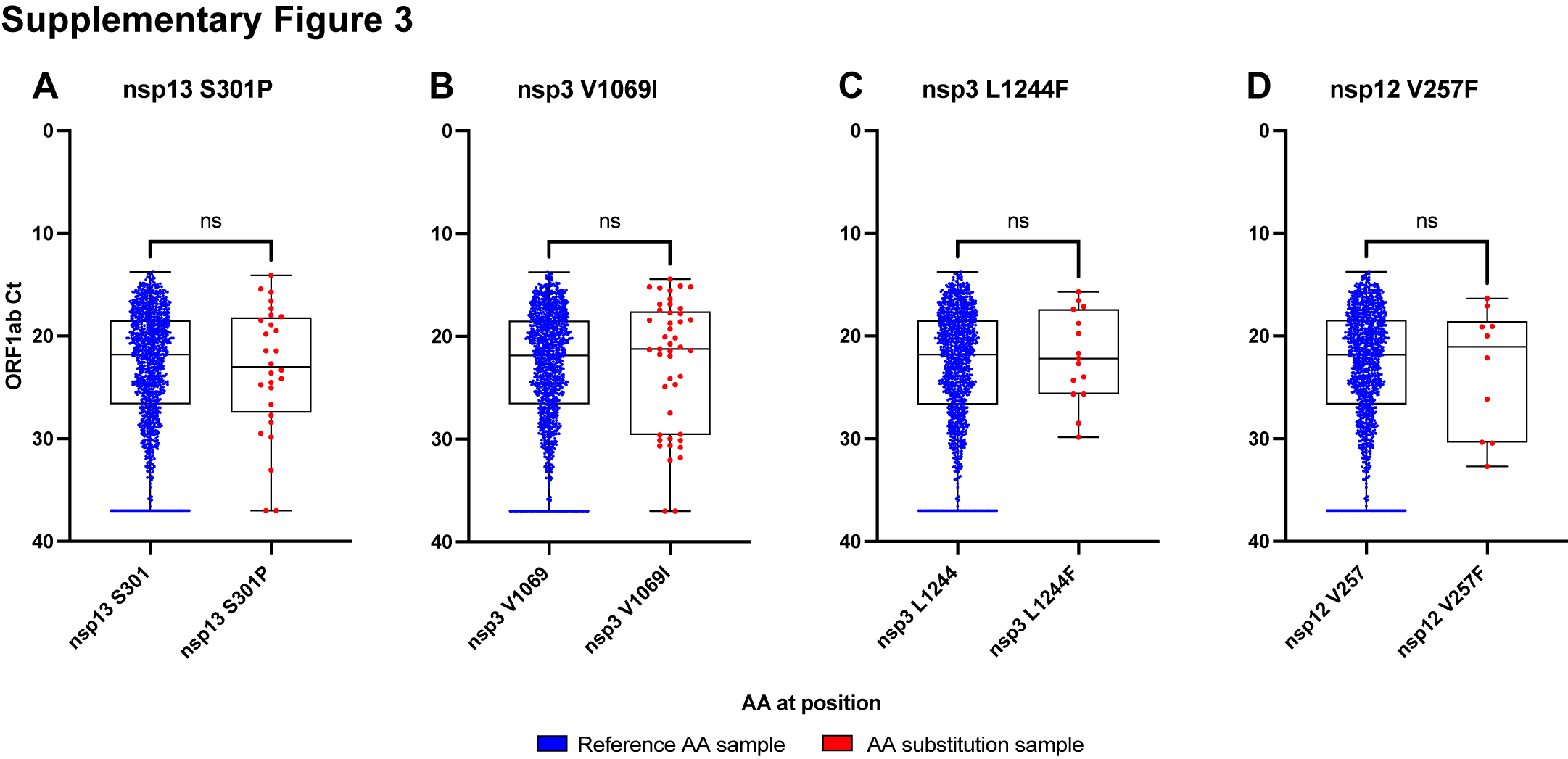

Supplement: FIG S3 [file mbio.03101-22-s0003.tif]
