# Supplementary material for: Baseline Sequencing Surveillance of Public Clinical Testing, Hospitals, and Community Wastewater Reveals Rapid Emergence of SARS-CoV-2 Omicron Variant of Concern in Arizona, USA
Source: mBio. 2023 Jan 9;14(1):e03101-22. doi: 10.1128/mbio.03101-22 (PMC9972916; doi:10.1128/mbio.03101-22)
Supplement: TABLE S1 [file mbio.03101-22-s0004.docx]

**Table S1: Variant lineages and specific mutations used to validate Freyja analysis**

| **Delta Lineages** |  | **Delta specific mutations** |
| --- | --- | --- |
| AY.103 |  | G4181T |
| AY.44 |  | C6402T |
| AY.39 |  | G9053T |
| AY.100 |  | A11201G |
| AY.3 |  | G15451A |
|  |  | C16466T |
|  |  | C19220T |
|  |  | C23604G |
|  |  | G24410A |
|  |  | C23604G |
|  |  | C25469T |
|  |  | T26767C |
|  |  | C27874T |
|  |  | G28881T |
|  |  | G28916T |
|  |  |  |
|  |  |  |
| **Omicron lineages** |  | **Omicron specific mutations** |
| BA.1.1 |  | A2832G |
| BA.1 |  | G8393A |
| BA.1.15 |  | C10449A |
| BA.1.20 |  | A11537G |
| BA.1.1.18 |  | C21762T |
|  |  | C23202A |
|  |  | C23525T |
|  |  | T23599G |
|  |  | C23604A |
|  |  | C24130A |
|  |  | A24424T |
|  |  | T24469A |
|  |  | C24503T |
|  |  | C26270T |
|  |  | G28881A |
|  |  | G28882A |
|  |  | G28883C |
