# Supplementary material for: Baseline Sequencing Surveillance of Public Clinical Testing, Hospitals, and Community Wastewater Reveals Rapid Emergence of SARS-CoV-2 Omicron Variant of Concern in Arizona, USA
Source: mBio. 2023 Jan 9;14(1):e03101-22. doi: 10.1128/mbio.03101-22 (PMC9972916; doi:10.1128/mbio.03101-22)
Supplement: TABLE S2 [file mbio.03101-22-s0005.docx]

**Table S2: Omicron RT-dPCR assay (Omn143) components and sequences**

| **Assay Component** | **Sequence** |
| --- | --- |
| Omn143-Fwd | 5’-GCTACTAATGTTGTTATTAAAGTCTGTGAATTTC-3’ |
| Omn143-Rev | 5’-GTGCAATTATTCGCACTAGAATAAACTC-3’ |
| Omn143 Probe | 5’-56-FAM/CATTTTTGGACCACA/3MGBEc/-3’ |
| Omicron BA.1 Control | 5’-CGCTGTAATACGACTCACTATAGGGTAATAAGAGGCTGGATTTTTGGTACTACTTTAGATTCGAAGACCCAGTCCCTACTTATTGTTAATAACGCTACTAATGTTGTTATTAAAGTCTGTGAATTTCAATTTTGTAATGATCCATTTTTGGACCACAAAAACAACAAAAGTTGGATGGAAAGTGAGTTCAGAGTTTATTCTAGTGCGAATAATTGCACTTTTGAATATGTCTCTCAGCCTTTTCTTATGGACCTTGAAGGAAAACAGGGTAATTTCAAAAATCTT-3’ |
| Delta Control | 5’-CGCTGTAATACGACTCACTATAGGGAGAGGCTGGATTTTTGGTACTACTTTAGATTCGAAGACCCAGTCCCTACTTATTGTTAATAACGCTACTAATGTTGTTATTAAAGTCTGTGAATTTCAATTTTGTAATGATCCATTTTTGGATGTTTATTACCACAAAAACAACAAAAGTTGGATGGAAAGTGGAGTTTATTCTAGTGCGAATAATTGCACTTTTGAATATGTCTCTCAGCCTTTTCTTATGGACCTTGAAGGAAAACAGGGTAATTTCAAAAA-3’ |
